# Editorial: Pulmonary Hypertension: Mechanisms and Management, History and Future

**DOI:** 10.3389/fmed.2020.00125

**Published:** 2020-04-21

**Authors:** A. A. Roger Thompson, Martin R. Wilkins, Jim M. Wild, David G. Kiely, Allan Lawrie

**Affiliations:** ^1^Department of Infection, Immunity and Cardiovascular Disease, The University of Sheffield, Sheffield, United Kingdom; ^2^Sheffield Pulmonary Vascular Disease Unit, Royal Hallamshire Hospital, Sheffield Teaching Hospitals NHS Foundation Trust, Sheffield, United Kingdom; ^3^Department of Medicine and National Heart and Lung Institute, Imperial College London, London, United Kingdom; ^4^POLARIS, Academic Unit of Radiology, Department of Infection, Immunity and Cardiovascular Disease, University of Sheffield, Sheffield, United Kingdom; ^5^INSIGNEO Institute for in silico Medicine, The University of Sheffield, Sheffield, United Kingdom

**Keywords:** pulmonary hypertension, screening tools, biomarkers, heart failure, therapeutics, imaging

From pulmonary hypertension observed at high altitude to pulmonary hypertension associated with left heart disease, this Frontiers Research Topic illustrates the diversity of clinical and pathological phenotypes associated with elevated pressure in the pulmonary circulation. The topic articles highlight the breadth of ongoing research into understanding this multi-faceted disease and are based on the content of a symposium that drew inspiration from the work of the British pathologist, Donald Heath, who published his manuscript on hypertensive pulmonary vascular disease after working as junior doctor in Sheffield (1, 2).

Despite advances in therapy over the last two decades, most forms of pulmonary hypertension (PH) have a poor prognosis. The greatest improvements in outcome have been observed in patients with CTEPH (3) where surgery may provide a cure, and pulmonary arterial hypertension (PAH) where younger patients with idiopathic disease (IPAH) in the UK now have a 5-year survival in excess of 80% (4). Intuitively, earlier diagnosis and institution of treatment may slow vascular remodeling and improve outcomes. However, despite increased awareness and the availability of new therapies the time from symptom onset to diagnosis remains unchanged at 2–3 years, suggesting that new approaches need to be taken to improve disease detection (5). This highlights the importance of identifying easily performed and scalable tests that can be deployed in the investigative pathway. Three of the studies in this Research Topic highlighted different modalities used in the diagnosis of pulmonary hypertension: exercise, serological biomarkers, and imaging. Billings et al. reported the utility of exercise testing in newly diagnosed, treatment naïve patients with pulmonary hypertension. Although this was a large retrospective study, only a small number of patients (1% of 895) were diagnosed while in WHO FC I. These data are consistent with early vascular changes preceding the development of symptoms and highlight the need for effective and sensitive screening tools for early disease. Billings et al. provide evidence that an incremental shuttle walk distance (ISWD) of <80% predicted identified 8 out of 9 patients with pulmonary hypertension in WHO FC I, while diffusing capacity (DLCO) measurements, using a cut-off of 80% predicted, only identified 4 of these patients. Further work is required to assess whether the simple and adaptable incremental shuttle walk test is a suitable field walking test to identify exercise limitation and to aid screening of high-risk populations, perhaps in combination with other biomarkers.

One such high risk group are those with connective tissue diseases, particularly systemic sclerosis (6). Hickey et al. discuss putative blood-based or lung tissue biomarkers for the identification of systemic sclerosis patients who have developed pulmonary hypertension. A significant issue with potential biomarkers is heterogeneity of patient phenotype even within this sub-group of PAH patients (7). Notably, the contribution of co-morbid interstitial lung disease is difficult to unravel. Hickey et al. also point out limitations associated with NT-proBNP, perhaps the foremost blood-based biomarker for PH in clinical use. While NT-proBNP features as an important component of early screening algorithms for SSc-PAH (8), it lacks specificity and carries the inherent disadvantage that the right ventricular strain, driving its release, is not a feature of early disease.

The third study with a focus on enhancing diagnosis, Chin et al., reports a strong correlation between CT measurement of pulmonary artery diameter and mean pulmonary artery pressure in the presence or absence of interstitial lung disease. This report implies that use of simple CT parameters could help predict presence of pulmonary hypertension in patients with lung disease, contrasting with previous reports suggesting that PA diameter was a less useful indicator of PH in patients with interstitial lung disease. Nonetheless, however useful our predictive tools, the armory of treatment of PH in these patients with interstitial lung disease remains frustratingly empty.

Work on the role of hypoxia in vascular remodeling offers potential promise for patients with PH associated with lung disease. At a molecular level, the importance of hypoxia inducible factors in vascular cell phenotypes is becoming clearer. These oxygen sensors, the discovery of which was recently acknowledged by the 2019 Nobel prize award to three HIF-biologists, are discussed in a review by Young et al. and the specific impact of altitude hypoxia on the pulmonary circulation is highlighted. While hypoxia has a defining role in PH associated with hypoxia and lung disease, the relevance of hypoxia at early stages of the pathogenesis of other forms of PH is less clear.

One important mediator of vascular remodeling with a potentially ubiquitous role across all forms of PH is neutrophil elastase. Neutrophils are considered the dominant source of elastase *in vivo* but as noted by Taylor et al., they are a relatively understudied cell in PH. Nonetheless, an excess of neutrophils (or high neutrophil to lymphocyte ratio) is reportedly correlated with poor outcomes in patients with PH (9) and inhibition of neutrophil elastase reversed disease in mouse models (10). The effect of elastase on turnover of the vascular extracellular matrix, and specifically changes resulting in stiffening of the large and small pulmonary vessels, is the subject of the review by Sun and Chan. Recent work from this group has shown that changes in arterial stiffness precede proliferative remodeling and that mechanosensitive pathways play important roles in regulating vascular cell expansion (11).

The complexity of pro-proliferative signaling in the pulmonary vasculature is further illustrated by the role of TRAIL, reviewed by Braithwaite et al.. Cleavage of TRAIL, a transmembrane protein, results in a soluble cytokine that can signal via a number of cell surface receptors but can also bind to decoy receptors. TRAIL canonically induces apoptosis in cancer cells and in virus-infected epithelial cells but paradoxically promotes vascular smooth muscle cell proliferation. These contrasting effects are mediated by at least two distinct signaling pathways and but exactly how cell and context-specific effects of TRAIL are mediated requires further study. Notably, the importance of TRAIL in infection, fibrosis and cancer might complicate therapeutic targeting of this pathway. However, other mediators known to interact with TRAIL, such as osteoprotegerin, have shown therapeutic promise (12).

Mechanistic insights are increasingly being generated by omics studies (13, 14) and this burgeoning field is reviewed by Hemnes. Blood-based or genetic signatures to enhance the granularity of patient classification or simply to facilitate diagnosis would have great utility, although relatively small patient numbers limit power and validation of predictive signatures. Furthermore, identifying whether omics are capable of detecting changes that precede pulmonary vascular remodeling will be challenging. Nonetheless, omics approaches could not only be used to stratify or diagnose PH but could also provide more personalized treatment approaches. Following interesting work on patients with positive vasodilator responses (15), Hemnes suggests using omics data to identify signatures defining a good response with other treatments. The concept of enriching clinical studies using specific omics signatures and then refining those signatures based on clinical response is proposed. Such strategies might increase the efficiency of later phase clinical trials.

While the majority of topic articles focus on the pulmonary circulation, Charalampopoulos et al. provide a comprehensive overview of PH due to left heart disease. Echocardiographic features that favor left heart disease over PAH are highlighted and the authors discuss studies reporting use of fluid challenges or exercise during right heart catheterization to identify occult left heart disease. The question of whether pulmonary vasodilators have any role in these patients or in patients with combined pre- and post-capillary pulmonary hypertension is the subject of ongoing clinical trials (e.g., SERENADE, NCT02246634) but unfortunately for this large group of patients, there is as yet, no clear answer.

The final review in the topic, by Middleton et al., notes the lack of specific guidelines for arrhythmia management in PAH. Indeed, there is a paucity of data on arrhythmia prevalence and on how frequently arrhythmia contributes to death in PAH patients. Deterioration in the context of atrial arrhythmia is a common clinical scenario that is potentially reversible. Therefore, exciting advances in monitoring technology, including wearable or implantable monitors, are an important avenue for future research. Indeed, our group are examining the utility of invasive pulmonary artery pressure and implantable rhythm monitors.

The broad and systematic approaches covered by the manuscripts included in this Research Topic highlight many of the current challenges to improve patient outcome and well-being in PH. Earlier diagnosis of patients with PH remains a major challenge. Despite advances in treatment and increasing awareness of the disease over the last 2–3 decades, many patients arrive at specialist centers after a significant diagnostic delay (16). While not reviewed in this topic, recent work from our center has sought to improve this issue by highlighting the potential use of artificial intelligence to interrogate routine healthcare data in order to identify patients at high risk of idiopathic PAH at an earlier stage (5). Furthermore, the topic only alludes to the use of novel tools to phenotype these high-risk patients. Multi-omic profiling and imaging of cardiac and lung structure and function (17, 18) including the use of emerging techniques such as hyperpolarised gases (19) or 4-dimensional magnetic resonance imaging (20) will likely hold the key to developing more sensitive and specific assessment tools. Such tools should generate new insights into molecular targets that alter vascular remodeling and could provide better clinical trial endpoints for assessment of treatment efficacy. The development of biomarkers and imaging tools using patients with earlier disease should be a priority for improving the treatment of PH in the precision medicine era.

## Author Contributions

AT and AL drafted the editorial with input from MW, JW, and DK.

## Conflict of Interest

The authors declare that the research was conducted in the absence of any commercial or financial relationships that could be construed as a potential conflict of interest.
